# Measurements of periodically perturbed dewetting force fields and their consequences on the symmetry of the resulting patterns

**DOI:** 10.1038/s41598-021-92544-x

**Published:** 2021-06-23

**Authors:** Konstantinos Roumpos, Sarah Fontaine, Thomas Pfohl, Oswald Prucker, Jürgen Rühe, Günter Reiter

**Affiliations:** 1 Institute of Physics, University of Freiburg, Hermann-Herder-Straße 3a, 79104 Freiburg, Germany; 2 Freiburg Materials Research Center, Stefan-Meier-Straße 21, 79104 Freiburg, Germany; 3 Freiburg Center for Interactive Materials and Bioinspired Technologies (FIT), University of Freiburg, Georges-Köhler-Allee 105, 79110 Freiburg, Germany; 4 Department of Microsystems Engineering, University of Freiburg, Georges-Köhler-Allee 103, 79110 Freiburg, Germany

**Keywords:** Surfaces, interfaces and thin films, Soft materials, Polymers

## Abstract

We studied the origin of breaking the symmetry for moving circular contact lines of dewetting polymer films suspended on a periodic array of pillars. There, dewetting force fields driving polymer flow were perturbed by elastic micro-pillars arranged in a regular square pattern. Elastic restoring forces of deformed pillars locally balance driving capillary forces and broke the circular symmetry of expanding dewetting holes. The observed envelope of the dewetting holes reflected the symmetry of the underlying pattern, even at sizes much larger than the characteristic period of the pillar array, demonstrating that periodic perturbations in a driving force field can establish a well-defined pattern of lower symmetry. For the presented system, we succeeded in squaring the circle.

## Introduction

In our study, we focused on the impact of periodic disturbances on breaking the symmetry of moving contact lines. In a huge number of materials and device fabrication processes, transport of materials on surfaces is directed by moving contact lines^1^. Typically, a locally initiated contact line will advance in the radial direction leading to structures of circular symmetry, allowing the description of movement by a single parameter, i.e., the radius of the hole and how it grows in time. This circular symmetry is rather stable even when the movement of the contact line is perturbed by frequent but random pinning events on rough or dirty surfaces. Therefore, one might expect that also periodic disturbances on the development of a moving contact line, such as in the dewetting of thin polymer films on arrays of deformable pillars, may not destroy circular symmetry. Dewetting is the process of retraction of a contact line leading to the removal of a fluid from a non-wettable surface. For a homogeneous thin polymer film, dewetting may start by random nucleation of holes of circular symmetry whose diameters increase with time. Dewetting has proven to be a valuable tool for investigating properties of thin polymer films (e.g. rheological properties, viscosity, residual stress) by simple experiments^2–6^. Dewetting has been used as a suitable tool for the formation of non-periodic patterns at a low cost, without the need for a lithographic process^7^. Using substrates with regular chemical^8, 9^ or topographical^10–13^ structures, ordered patterns could be obtained. However, the emerging dewetting patterns often only reproduce the template of the imprinted surface pattern. It is still unclear, if the symmetry of the underlying substrate pattern can be retained, when the contact line moves across multiple elements of the pattern.

Extensive research has been performed also on dewetting of freely standing films. In freely standing viscous films, the radius of dewetting holes was found to increase exponentially with time without loss in circular symmetry^14^. Remarkably, contrary to supported films, no rims were formed at the border of the dewetting holes. The removed material was not accumulated close to the moving contact line like for supported films but was evenly distributed within the film^15–18^. Dewetting was also examined for polymer films that were transferred on arrays of widely separated stiff pillars, which did not deform under the action of capillary forces. In those studies, rather thin films were used with correspondingly small distances between large numbers of frequently coalescing holes. Thus, the focus of those studies was mainly on the latest stages of dewetting of unperturbed freely standing polymer films^14, 19, 20^. Moreover, spreading of liquids on patterned solid substrates was examined rigorously by many scientists. When spreading a liquid droplet on such patterned substrates, its shape can deviate from its initial shape of a spherical cap. Eventually, the shape of the contact line of the droplet may adapt the symmetry of the underlying pattern^21–23^.

**Figure 1:**
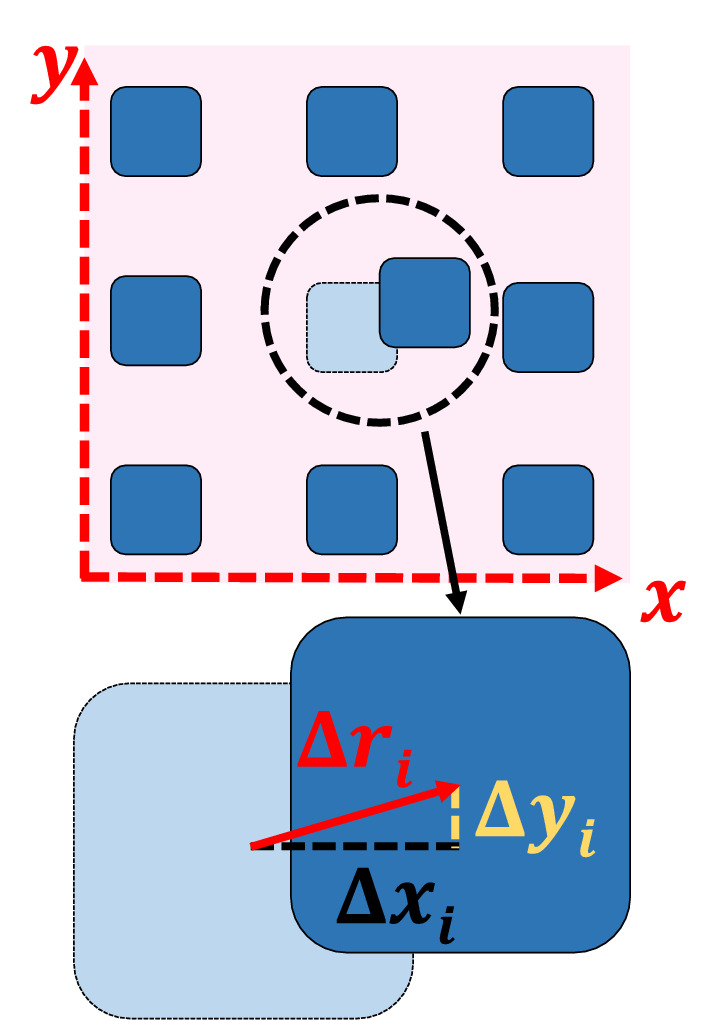
Pillars acting as force sensors. Sketch of the top view of the pillar array. One of the pillars has been deflected from its initial position. Knowing the modulus of the pillar and its size, the deflection $\Delta \vec{r}_{i}$ can be translated into force components acting on pillar $i$ in *x*- and *y*-direction.

In our study, we suspended polymer films on periodic arrays of rather closely spaced and *deformable* micro-pillars, whose deflections are directly proportional to the forces that are acting upon them. Using optical microscopy, we followed dewetting in time and observed periodic perturbations exerted by the pillars on the moving contact line of dewetting holes. Simultaneously measuring deformations of individual pillars allowed determining the force fields around and in the vicinity of dewetting holes (see Fig. 1). Similar arrays of deformable micro-pillars are commonly used for biophysical investigations on cell adhesion where forces exerted due to cell movement and migration can be determined^24–27^. Here, we employ such pillar arrays as a novel approach for an accurate and consistent determination of the traction forces exerted by a dewetting polymer film. Our approach allows visualizing and precisely measuring the two-dimensional force field responsible for the flow of polymer away from a dewetting hole. We also examined the shape of the contact line of the dewetting hole as it was perturbed by the underlying pillars.

## Results and discussion

By heating thin polystyrene films suspended on arrays of pillars to temperatures above the glass transition temperature ($T_{{\text{g}}}$) of the polymer, the formation of dewetting holes was initiated at random positions^28^. Consistent with previous studies^6^, the nucleation density of dewetting holes decreased with increasing film thickness. We examined two regimes of dewetting, distinguished by the radius $R$ of the dewetting holes being comparable or much larger than $~\omega$, the periodicity of the square array of pillars. For the early stage of dewetting ($R~ < ~2\omega$), we visualized and measured the force field acting at the dewetting front. For these experiments, we used films of 50 nm thickness annealed at 115 °C (slightly above $T_{{\text{g}}}$). For the later stages of dewetting ($R > 2\omega$), we used thicker films of about 400 nm thickness annealed at 180 °C. In this case, fewer holes were nucleated and thus large values of $R$ could be achieved before neighboring dewetting holes coalesced. For these larger holes, we examined mainly the shape of the envelope for large diameters ($R~ > ~8\omega$).

### Early stage of dewetting: hole nucleation and initial growth

**Figure 2:**
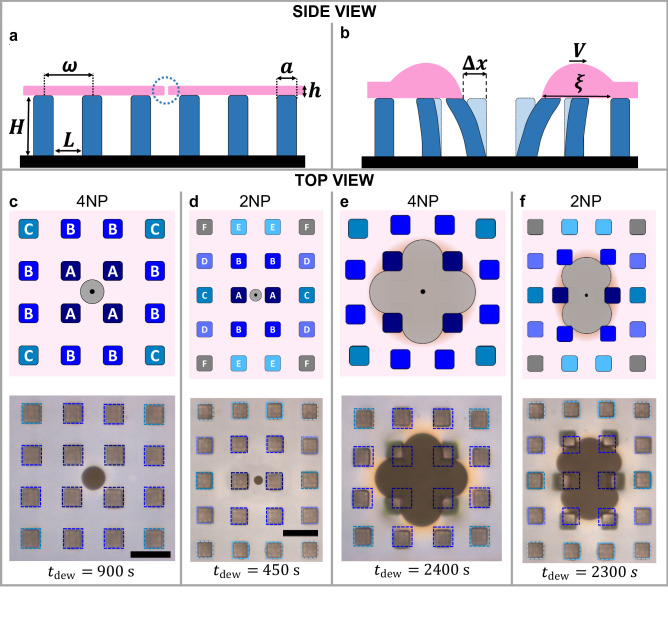
Breaking circular symmetry by locally pinning the contact line of dewetting holes. Deformation of pillars during the growth of dewetting holes in a thin polystyrene film of thickness $~h$ suspended on an array of pillars (characterized by the width $a$, the height $H$ and separated by $~L$, yielding the period $\omega$). (**a**) Scheme indicating the nucleation of a dewetting hole (inside the dotted circle). (**b**) As dewetting advances, pillars touching the contact line of a dewetting hole, as well as more remote pillars, become deformed by ${\Delta}x$ due to the flow of polymer. The dewetting process is accompanied by the formation of a rim (characterized by the width $\xi$) around the holes. (**c**) and (**d**) Sketches and corresponding optical micrographs of an initially circular dewetting hole for scenarios 4NP and 2NP, respectively. In the sketches, the dewetting hole is shown in grey with a black dot in its center representing its point of nucleation. (**e**) and (**f**) show sketches and corresponding optical micrographs at later stages of dewetting for scenarios 4NP and 2NP, respectively. The blue squares in the optical micrographs indicate the initial positions of the pillars. The black bars represent a length of 10 μm.

At the very beginning, all dewetting holes had circular symmetry without any detectable rim around them. However, when their contact line approached a pillar, the shape of the holes became distorted. At the same time, a rim started to form around the holes (Fig. 2). Regarding to the origin of the dewetting hole with respect to the positions of the nearest pillars, we distinguished two different scenarios. For a scenario abbreviated 4NP in the following, a dewetting hole was nucleated at almost equal distance to four Nearest (neighboring) Pillars. For scenario 2NP, the hole was nucleated at almost equal distance between two Nearest Pillars. In our experiments, we have analyzed shapes of several hundreds of nucleated dewetting holes. The behavior of all analyzed holes is covered by the scenarios of the 4NP and 2NP holes, for which we have analyzed the temporal evolution of hole diameter and the distortion of the contact line impacted by the deformable pillars of the course of the hole growth in great detail. The presented analysis can also be carried out for randomly nucleated dewetting holes, yielding the same conclusions.

#### 4NP: dewetting hole nucleated in between 4 pillars

For the 4NP case, holes showed characteristic changes in shape as presented in Fig. 2 (see also Supplementary Movie 1). When the contact line of these holes approached the four pillars, its circular shape became distorted, resembling eventually a square hole with rounded corners. Interestingly, the orientation of this square was tilted by 45° with respect to the *x*- and *y*-axes (as defined in Fig. 1). The distortion was the consequence of pinning of the contact line at the pillars causing a restriction of polymer flow at these positions. In conjunction with the distortion of the contact line, a halo around the dewetting hole became visible in the optical micrographs, indicating the build-up of a rim. As the hole grew larger, the contact line of the dewetting hole remained pinned at the pillars, causing a deformation of the pillars. As a consequence, the shape of the hole became distorted further, accompanied by the formation of a larger and clearly visible rim (Fig. 2). The deformation of four nearest pillars increased with increasing dewetting time. We note that the width, $\xi$, of the rim were not the same at all positions along the contact line of the dewetting hole. In the vicinity of pillars, the shape of the rim (indicated in the micrographs by variations in the interference colors) varied strongly. Radial polymer flow within the rim caused deformations of pillars even at some distance to the contact line. The degree of deformation was smaller for a larger distance between contact line and pillar. Pillars at distances larger than $\xi$ did not show any deformation.

For further analysis, we categorized the pillars in three groups (A, B and C) based on their distance from the center of the dewetting hole, *s*_J_(4NP), which is related through $R$ to the distance to the contact line. Distances *s*_J_(4NP) for three categories of pillars near the contact line are presented in the Supplementary Fig. 1, labeled by the index “J” being A, B or C. In Fig. 2, these three groups of pillars are shown in different shades of blue. Group A comprised the pillars nearest to the dewetting hole, which were the first ones to become deformed. Group B comprised the next nearest pillars, which started to deform when the rim of the dewetting hole approached them. Group C comprised pillars at larger distances (shown in the corners of the image). These pillars only exhibited slight deformations during the explored duration of the experiment.

#### 2NP: dewetting hole nucleated between two pillars

In Fig. 2, we show also characteristic changes in the shape of a hole of the 2NP case (see also Supplementary Movie 2) with the initially circular hole becoming deformed earlier than in the 4NP case. When the contact line became pinned at the two nearest pillars, the deflections of these pillars were increasing in directions opposite to each other. The shape of the contact line of the dewetting hole diverged progressively more from the initial circle, yielding a shape resembling a stadium, characterized by two parallel long straight parts connected at their ends by half circles. Later on, the contact line of the dewetting hole approached the four next nearest pillars, which started to deflect as well. Similar to the 4NP scenario, when the pillars perturbed the polymer flow, a rim formed around the hole.

For the 2NP case, we categorized the pillars in six groups (A, B, C, D, E and F) depending on their distance from the center of the dewetting hole, *s*_J_(2NP) with the index J running from A to F (see Supplementary Fig. 1). Group A comprised only the two pillars nearest to the dewetting hole, which deform first. Group B comprised the next nearest pillars, which started to deform when the dewetting hole approached them. If the contact line was pinned in the direction of the $x$-axis, holes grew more rapidly in the orthogonal direction of the $y$-axis. Groups C, D and E comprised intermediate pillars at progressively increasing distance from the center of the dewetting hole, which became deflected only at slightly later stages of dewetting. The most distant pillars of group F (labeled grey in Fig. 2) did not show any deflection during the explored duration of the experiment.

#### Dewetting dynamics and force field mapping

**Figure 3:**
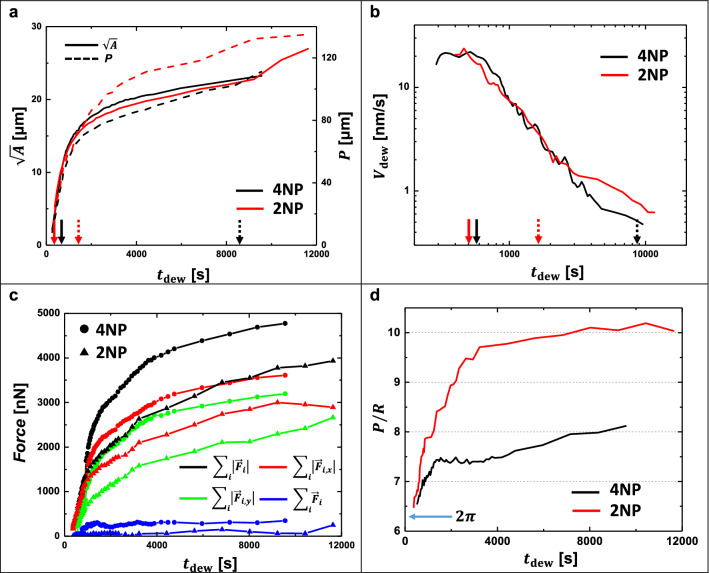
Changes in dewetting dynamics are accompanied by changes in the forces acting on the pillars. (**a**) Square root of the area $\sqrt A$ and the perimeter$~P~$ of dewetting holes as a function of dewetting time $t_{{{\text{dew}}}}$. (**b**) Double logarithmic plot of mean dewetting velocity $V_{{{\text{dew}}~}}$ as a function of $t_{{{\text{dew}}~}}$. The arrows on the horizontal axis indicate the positions where the contact line of the dewetting holes first touched the pillars of group A (solid arrows) and group B (dashed arrows). (**c**) Graph of various sums of forces acting on pillars for 4NP and 2NP scenarios. (**d**) The perimeter $P$ of the dewetting hole divided by its effective radius $R$ as a function of $t_{{{\text{dew}}}}$, showing the progressive deviation from its initial circular shape.

We followed the growth of dewetting holes by determining the square root of the area of the dewetting hole $\left( {\sqrt A } \right)$ and their perimeters ($P$) as a function of time (see Fig. 3a). We defined a mean dewetting velocity ($V_{{{\text{dew}}~}}$) as $V_{{{\text{dew}}}} = \sqrt A {\text{/}}\Delta t$. Initially, when polymer flow was not yet restricted by pillars (Fig. 3b), $V_{{{\text{dew}}~}}$ was large and even indicated a tendency to increase. However, once the contact line of the dewetting hole approached the nearest pillars, $V_{{{\text{dew}}~}}$ decreased in time. Slowing down of $V_{{{\text{dew}}~}}$ was accompanied, as described earlier, by a change from the initial circular symmetry of the dewetting holes to a shape of “rounded squares” or “stadiums” for 4NP and 2NP holes, respectively.

The corresponding changes in the values of the force fields acting on the pillars are given in Fig. 3c. Forces were derived from the deflection of each pillar (see Eq. (3) given in the “Methods” section). We measured the sums of the lengths of the $~x$- and $y$-components of the force vector $\vec{F}_{i}$ of each pillar $i$, $\mathop \sum \limits_{i} F_{{i,x}}$ and $\mathop \sum \limits_{i} F_{{i,y}}$, respectively, as well as the total force, $\mathop \sum \limits_{i} \left| {\vec{F}_{i} } \right|$. Here, $i$ covered the range $1 \le i \le 16$ as we have 16 pillars for 4NP and only 16 out of the 20 pillars of 2NP exhibit deflections. In all cases, as the pillars started to deflect, $\mathop \sum \limits_{i} \left| {\vec{F}_{i} } \right|$ increased with $t_{{{\text{dew}}~}}$. It is interesting to see that the sum of all force vectors (i.e., $\mathop \sum \limits_{i} \vec{F}_{i}$) was always close to zero, $\mathop \sum \limits_{i} \vec{F}_{i}$ ≈ 0, implying that the forces acting at the contact line of the dewetting hole “pushed” on average uniformly in all directions.

The perimeter $P~$ of the dewetting hole divided by its effective radius $R = \sqrt {\frac{A}{\pi }}$ is given in Fig. 3d. For a circular geometry a value of $2\pi$ is expected ($2\pi R~/~R$), which is true for both 4NP and 2NP scenarios at the beginning of the experiment. As the dewetting holes kept growing, for 4NP, the approximate boundary of the hole resembled a square indicated by the ratio $P/R$ approaching a value of about 8. For 2NP, the approximate boundary of the hole resembled a rectangle, indicated by the ratio $P/R$ approaching a value of about 10.

**Figure 4:**
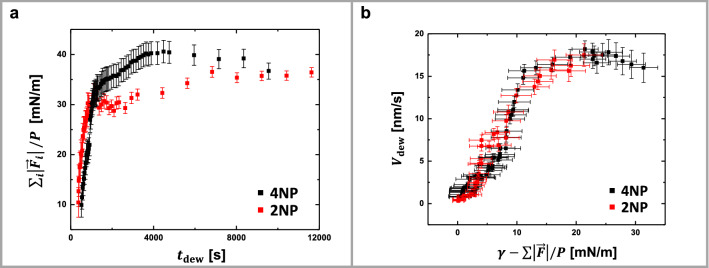
The dewetting velocity approached zero when elastic forces (visualized by the deformations of the pillars) acting at the contact line balanced capillary forces. (**a**) Mean tension, $\mathop \sum \limits_{i} \left| {\vec{F}_{i} } \right|/P$, as a function of $t_{{{\text{dew}}~}}$. (**b**) $V_{{{\text{dew}}}}$ as a function of the sum of opposing capillary and elastic forces, $\gamma _{{{\text{PS}}}} ~{-}~\mathop \sum \limits_{i} \left| {\vec{F}_{i} } \right|/P~$.

As the dewetting film interacts with the surface of the pillars, the pillars are becoming deformed by opposing the driving force of dewetting. Dividing the sum of the absolute values of the forces on all pillars by the perimeter of the dewetting hole, $\mathop \sum \limits_{i} \left| {\vec{F}_{i} } \right|/P$, resulted in a mean tension acting on pillars that increased with $t_{{{\text{dew}}}}$ and thus with $R/\omega ~$ (Fig. 4a and Supplementary Fig. 2). Interestingly, for both scenarios, 4NP and 2NP, this tension approached for $V_{{{\text{dew}}}} \to 0$ (long $t_{{{\text{dew}}}}$) the value of the surface tension of polystyrene, $~\gamma _{{{\text{PS}}}}$:

1 $$\mathop {\lim }\limits_{{V_{{dew}} \to 0}} \frac{{\mathop \sum \nolimits_{i} \left| {\vec{F}_{i} } \right|}}{P} \approx \gamma _{{{\text{PS}}}} ,$$

with $$\gamma _{{{\text{PS}}}} \approx 34\;{\text{mN/m}}$$ at 115 °C^29^.

Dewetting is driven by forces acting at the contact line. In our system, the capillary force $\vec{F}_{{{\text{cap}}}} \left( {\gamma _{{PS}} } \right)$ (characterized by $\gamma _{{{\text{PS}}}}$) tries to enlarge the dewetting hole while the elastic response of the pillars $\vec{F}_{i}$ (which is proportional to the deflection $\Delta \vec{r}_{i}$; Fig. 1) opposes dewetting:

2 $$\vec{F}_{{{\text{dew}}}} = ~\vec{F}_{{{\text{cap}}}} \left( {\gamma _{{PS}} } \right) - \vec{F}_{i} \left( {{\text{}}\Delta \vec{r}_{i} } \right).$$

Most of the capillary energy is dissipated in the dewetting film proportional to its viscosity. Dissipation at the pillar-film interface due to friction is present as well. However, in our analysis we considered the pillars as “pinning sites”, assuming no sliding of the film over the pillars (thus no friction). Based on this assumption, we reached a maximum deformation of the pillars proportional to the driving force.

When the deformations of the pillars became so strong that the corresponding tension opposed the total capillary force, i.e., $\gamma _{{{\text{PS}}}} ~{-}~\mathop \sum \limits_{i} \left| {\vec{F}_{i} } \right|/P~ \to ~0$, dewetting came to a (temporal) halt, i.e., $V_{{{\text{dew}}}} ~ \to ~0$, as can be seen in Fig. 4b.

#### Forces on individual pillars

When the contact line of a dewetting hole approached a pillar, flow of the polystyrene film was opposed by frictional and elastic forces induced by the pillars. Consequently, a rim of accumulated polymers formed at the edge of the hole. The force field generated by polymer flow in the moving rim caused deformations of the pillars even before the contact line of the dewetting hole reached them. The force ($\vec{F}_{i} \left( {\Delta \vec{r}_{i} } \right)$) on the pillars closest to the contact line of the dewetting hole (group A) increased quickly. Initially, pillars further away from the dewetting hole experienced less force.

**Figure 5:**
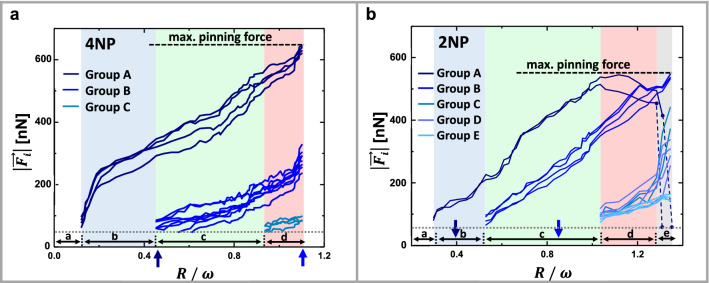
Mapping the force fields: force $\left| {\vec{F}_{{\text{i}}} } \right|$ acting on individual pillars as a function of $R/\omega .$ (**a**) For the 4NP scenario and (**b**) for the 2NP scenario. The arrows on the horizontal axis indicate the positions where the contact line of the dewetting holes first touched the pillars of group A and group B. The unpinning transition is indicated by blue dashed lines in b. The resolution of the pillar deflection was about 0.3 μm, corresponding to a minimum measurable force of about 50 nN, indicated by a grey dotted line.

To analyze the force fields and the impact of the underlying pillar array during dewetting of the suspended polystyrene film, we plotted the evolution of the deformation of the individual force sensing pillars as a function of $R/\omega$ (Fig. 5).

As the dewetting holes were growing, i.e., for increasing $R/\omega$, we observed that the individual force sensing pillars passed through a sequence of characteristic regimes. These regimes are indicated as separate regions of interest in the graphs of Fig. 5, represented by different hues of vertical shading (blue, green, red and grey) and by the lowercase labels at the bottom of Fig. 5a, b.

**Figure 6:**
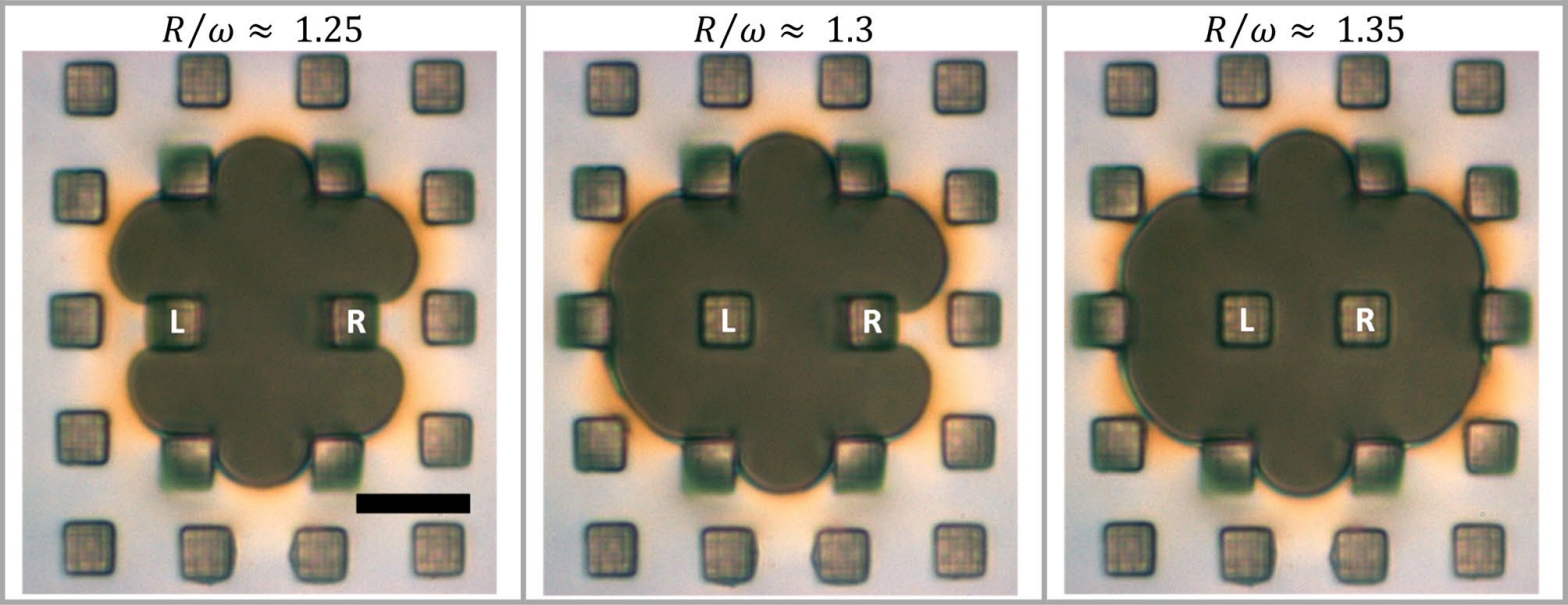
Unpinning and snapping back of pillars. Notice the increased deflection of the neighboring pillars each time a pinned pillar was released and jumped back to its initial position. Due to high nucleation density and corresponding frequent coalescence of dewetting holes, we observed unpinning events only for group A pillars in the 2NP scenario. The labels L and R stand for “left” and “right” pillar. At $R/\omega \approx$ 1.25: no unpinning of pillars yet. At $R/\omega \approx$ 1.30: unpinning of pillar L. At $R/\omega \approx$ 1.35: additional unpinning of pillar R. The length of the black bar represents 10 μm.

- $0 < ~R/\omega < 0.1$ for the 4NP and $0 < ~R/\omega < 0.2$ for the 2NP scenario (white sector):
  The hole grew without being perturbed by pillars and without a visible formation of a rim. No deflection of the pillars could be observed, meaning that no forces were acting on any of the pillars. $V_{{{\text{dew}}}}$ (Fig. 3b) was high and tended to increase slightly with $R/\omega$, as observed for dewetting in freely standing polystyrene films^14, 16^.
- $0.1 < ~R/\omega < 0.45$ for the 4NP and $0.2 < ~R/\omega < 0.5$ for the 2NP scenario (blue sector):
  The nearest pillars (group A) started to deflect progressively with increasing $R/\omega$, accompanied by a decrease in $V_{{{\text{dew}}}}$ (Fig. 3b). The formation of a rim around the holes was observed where polymers from the dewetted hole were accumulated (Fig. 2).
- $0.4 < ~R/\omega < 0.95$ for the 4NP and $0.5 < ~R/\omega < 1$ for the 2NP scenario (green sector):
  In addition to pillars of group A, intermediate pillars of group B started to deflect when the dewetting rim reached them. In this region, $V_{{{\text{dew}}}}$ decreased further (Fig. 3b).
- $R/\omega > 0.95$ for the 4NP and $1 < R/\omega < 1.25$ for the 2NP scenario (red sector):
  Additional pillars of group C for 4NP and groups C, D and E for 2NP started to deflect due to polymer flow in the dewetting rim. At $R/\omega \approx$ 1, pillars closest to the dewetting hole showed a maximum deflection, i.e., exerted a maximum pinning force of $\vec{F}_{{{\text{max}}}} = (600{\text{~}} \pm {\text{~}}50)\;{\text{nN}}$. We indicated $\vec{F}_{{{\text{max}}}} ~$ by black horizontal dashed lines in Fig. 6. Notice that for the pillars of group A in the 2NP scenario, $\vec{F}_{i}$ started to decrease slightly at $R/\omega \approx$ 1.15, i.e., the deflection of these pillars decreased slightly. Simultaneously, the contact line of the dewetting hole squeezed in between pillars and $\vec{F}_{i} ~$ for pillars of group B increased until $\vec{F}_{{{\text{max}}}}$ was reached (see also Supplementary Figs. 3 and 4b).
- $R/\omega > 1.25$ only for 2NP scenario: unpinning of the two nearest pillars (grey sector).

Due to the deformation of the pillar, elastic energy is stored in the pillar. When the total driving force becomes larger than the pinning force, the contact line detaches from the pillar and proceeds rapidly as the pillar snaps back to its undeformed state. The thereby released elastic energy is dissipated in the pillar. Upon unpinning, the extra capillary energy resulting from a deformation of the pinned contact line (the deviations from a circular hole) is dissipated due to the viscosity of the film.

The capillary force previously exerted on pillars of group A is now redistributed to the other pillars, especially to the next nearest ones (groups B and C), whose deflection increased significantly, as one can also see in Supplementary Fig. 5. This process is presented in detail in Fig. 6, where we observed the unpinning of pillars of group A (named L and R to distinguish the left and right pillar). Pillar L was released at $R/\omega \approx 1.3$ and pillar R at $R/\omega \approx 1.35$. In conjunction, we observed the straightening of the contact line accompanied by a strong increase of the dewetted area without a significant increase of the perimeter $P$ of the dewetted hole. This abrupt change is also indicated in Fig. 3a for $t_{{dew}} > 9000\,{\text{s}}$, where $\sqrt A$ increased while $P$ practically remained constant.

Due to the large number of dewetting holes generated in films of 50 nm, dewetting holes began to coalesce already for $R/\omega ~ \ge 1.5$, prohibiting further measurements on individual holes (Supplementary Fig. 5).

### Late stages of dewetting: “squaring the circle”

In order to examine the question of what will be the shape of the envelope of the contact line of dewetting holes when they grow larger, we reduced the number density of dewetting holes in the film. Thus, holes could grow to larger sizes before they coalesced with other holes. This scenario was achieved by using thicker polystyrene films of about 400 nm. However, if we had used the same $T_{{{\text{dew}}}} = 115\,^\circ {\text{C}}$ as for the previously discussed experiments, the correspondingly slow $V_{{{\text{dew}}}}$ would have caused extremely large dewetting times, $t_{{{\text{dew}}}}$ (several days). Therefore, we accelerated dewetting by performing experiments on these films at a higher $T_{{{\text{dew}}}} = 180~^\circ {\text{C}}$, where the viscosity of the employed atactic polystyrene was lower^30^, yielding higher values of $V_{{{\text{dew}}}}$.

**Figure 7:**
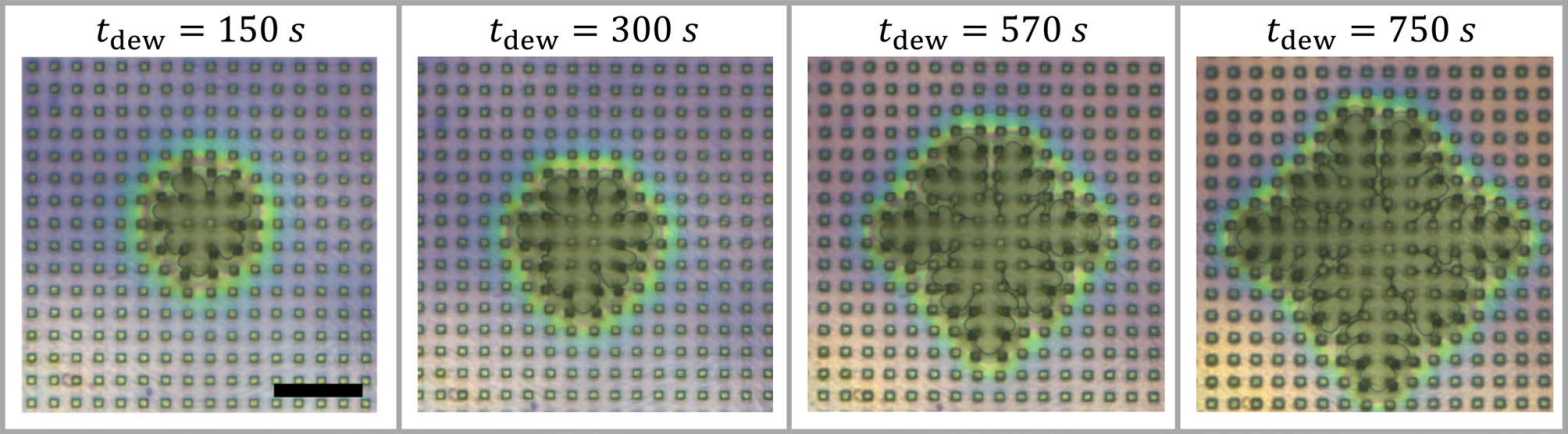
“Squaring the circle”. Dewetting of a 400 nm thick film of polystyrene performed at $T_{{{\text{dew}}}}$ = 180 °C on top of an array of deformable pillars for different values of $t_{{{\text{dew}}}}$. The length of the black bar represents 50 μm.

**Figure 8:**
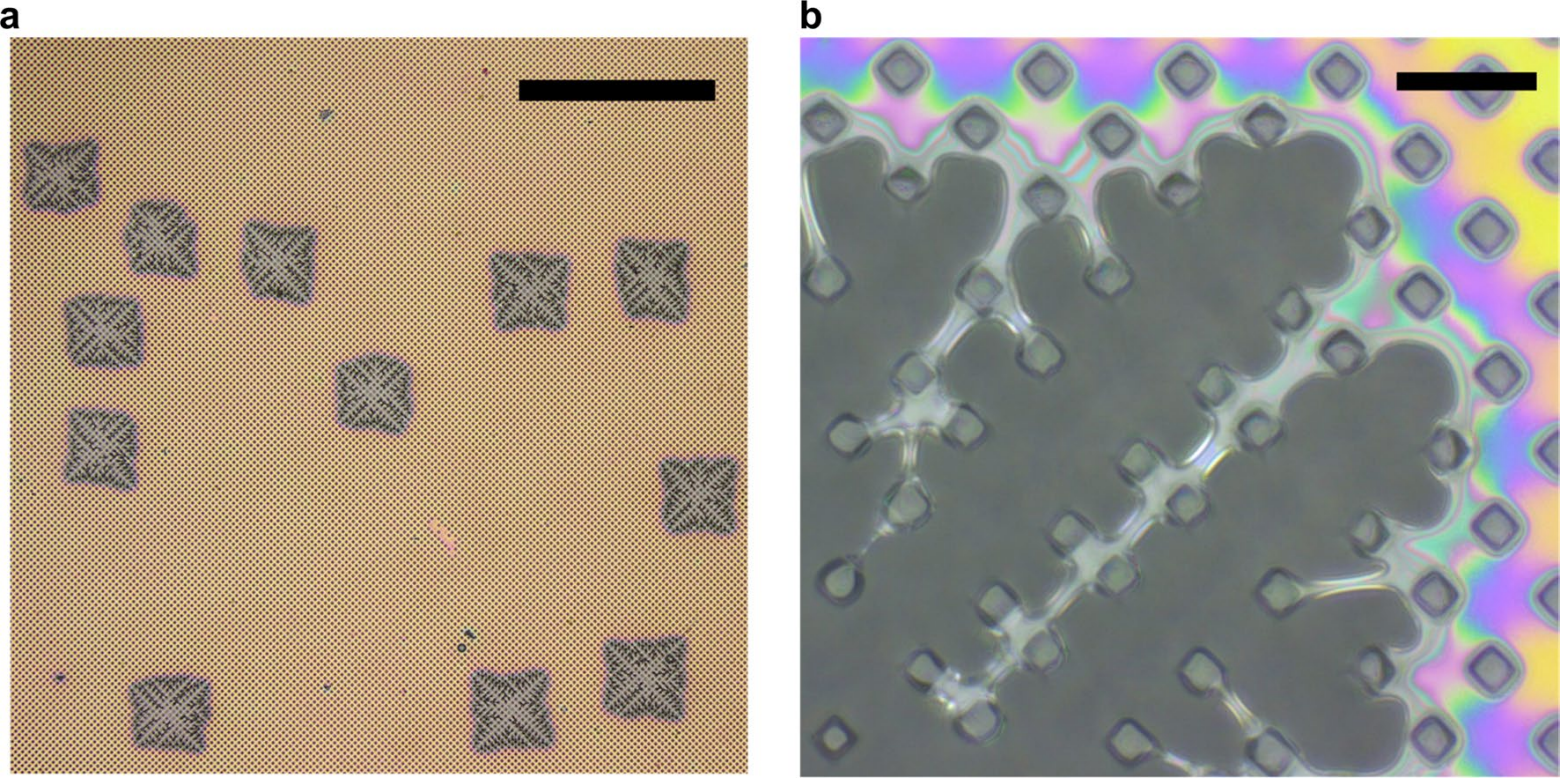
Uniquely oriented square dewetting patterns of almost uniform size. Optical micrographs taken at room temperature for the sample shown in Fig. 8 after $t_{{{\text{dew}}}} = ~25\;{\text{min}}$. The sample was rotated 45° in respect to the orientation (coordinate system) defined in Fig. 1. (**a**) Randomly distributed but uniquely oriented large dewetting holes showing that the envelope of the contact line of all dewetting holes had a square shape. Length of black bar: 500 μm. (**b**) Magnified upper right corner of one of the dewetting holes showing the formation of bridging filaments of polystyrene mainly in the directions of the diagonals of the pattern. Length of black bar: 20 μm.

Intriguingly, the envelope of the contact line of the dewetting holes showed nearly circular symmetry (in particular the rim represented by the greenish interference color shown in Fig. 7) even up to radii larger than ca. three times the period of the array of pillars. However, at much later stages, the envelope of the contact line of all dewetting holes approached a square shape, reflecting the symmetry of the underlying array of pillars. We note that the square was tilted by 45° with respect to $x$- and $y$-directions (as they are defined in Fig. 1). All dewetting holes showed this transition from the initially circular the square shape, independent of the position of their initial nucleation (Fig. 8a). Furthermore, all square dewetting holes had the same orientation with respect to the underlying pattern and were of approximately the same size, indicating that the dewetting dynamics and the corresponding force fields were the same for all dewetting holes. Accordingly, we conclude that eventually the symmetry of the substrate pattern was transferred to the envelope of the contact line of all dewetting holes. In the higher magnification image of Fig. 8b, we show that this transition involved the formation of bridging filaments of polystyrene between neighboring pillars mainly in the directions of the diagonals of the square dewetting pattern.

## Conclusions

Deformable pillars were utilized as precise sensors for measuring force fields generated during dewetting of polystyrene films. In the course of dewetting, the surrounding pillars impeded unperturbed polymer flow, leading to the distortion of the initially circular contact line of nucleated holes accompanied by the formation of a rim of accumulated polymers around these holes. The dewetting velocity decreased as the pillars became progressively more deformed. The force field also decayed for increasing distance from the dewetting hole. Analyzing the force field generated by the dewetting hole, we found that the sum of the absolute values of all forces acting on the pillars was proportional to the perimeter, yielding a constant measured tension per unit length of perimeter. This measured tension was identical to the value the surface tension of the dewetting polymer. Most intriguingly, the envelope of the contact line of dewetting holes in thick films showed a clear transition from an initially circular to a quadratic symmetry for hole diameters significantly larger than the period of the underlying substrate pattern. Thus, we can transfer the symmetry of this pattern to the envelope of the contact line of all dewetting holes. The strategy of directing moving contact lines by periodic disturbances represents a promising approach for controlled material transport and the design of adaptive and programmable materials surfaces.

## Methods

### Dewetting experiments

Isothermal dewetting of the suspended thin polystyrene films, i.e., the emergence and growth of holes, was induced by heating the samples above $T_{{\text{g}}}$ on a hot stage (Linkam, UK). Dewetting was followed in situ and in real time by optical microscopy (Zeiss AxioScope A1, 100 × objective). In order to measure the force field generated by a dewetting hole, experiments were performed on 50 nm films at $T_{{{\text{dew}}}} = 115\;^\circ {\text{C}}$. In order to examine the pattern formation during the late stage of dewetting, we used films of 400 nm thickness so that the number density of dewetting holes was reduced and the holes could grow larger before coalescing with other holes. These experiments were performed at $T_{{{\text{dew}}}} = 180\;^\circ {\text{C}}$.

For the experiments, we distinguish the following time scales: (i) We define the time for which the sample was kept on the hot stage, measured from the time the sample was placed on the hot stage, as the annealing time ($t_{{{\text{ann}}~}}$). (ii) The time, which passed from the placement of the film on the hot stage until a dewetting hole was nucleated, is called incubation time ($t_{{{\text{inc}}~}}$) of the dewetting hole. (iii) The duration of dewetting, measured from the moment a given hole was nucleated, is referred to as dewetting time ($t_{{{\text{dew}}~}}$). These time scales are related according to the equation, $t_{{{\text{dew}}~}} ~ = ~t_{{{\text{ann}}~}} {-}~t_{{{\text{inc}}~}}$. Thin polystyrene films that are prepared by spin coating contain residual stresses. It has been shown by dewetting experiments that these stresses relax progressively as the film is annealed at temperatures above $T_{{\text{g}}}$, leading to changes in viscoelastic behavior^31–33^. $t_{{{\text{inc}}~}}$ is therefore an important parameter that affects the dewetting dynamics and the rheological behavior of polystyrene films.

While holes were nucleated at different times after placing the film on the hot stage ($t_{{{\text{inc}}~}}$), we have only chosen holes for our experiments which were nucleated with $~t_{{{\text{inc}}}} < 2{\text{~s}}$, i.e., almost immediately after the sample was placed on the hot stage. We have captured a series of images at a time resolution of 1 s with a CCD camera (Zeiss 105 color).

Analysis of the micrographs was carried out using the image analysis software ImageJ (Supplementary Fig. 6). From this analysis, we determined the area, the perimeter and the radius of the dewetting holes, the deflection of the pillars and the distance of each pillar from the dewetting rim.

### Formation of pillar arrays

Deformable micro-pillars were fabricated on glass substrates using a lithographic process^34^. The material used for fabricating the pillars was a copolymer of 95% poly(*n*-butyl acrylate)**,** PnBA with 5% methacryloxybenzophenone, (MABP) ($M_{w} ~ =$ 144 kg mol^−1^, *Đ* = 2.48), with an elastic modulus of $E = ~$ 3.3 MPa and glass transition temperature of $T_{{\text{g}}}$ =  − 42 °C. The pillars had a square cross-section of $~a^{2}$ = 5 × 5 μm^2^, a height of $H$ = 15 μm and were separated by an empty space with a distance of *L* = 5 μm (in some cases, we used pillars of *L* = 5 μm) between them. The periodicity $\omega$ of the pattern was therefore $\omega$ = 10 μm (or 12 μm), from the center of one pillar to the center of the next one. These deformable pillars were used as force sensors. The deflection $\Delta r_{i} \left( {x_{i} ,y_{i} } \right)$ at the top part of pillar *i* was proportional to the force $F_{i}$ acting on it, according to the following equation^35^ (Hooke’s law):

3 $$F_{i} \left( r \right)~ = ~\frac{{Ea^{4} }}{{4H^{3} }}\Delta r_{i} \left( {x_{i} ,y_{i} } \right)$$

The total deflection $\Delta r_{i} \left( {x_{i} ,y_{i} } \right)$ was measured directly from the micrographs. Possible twisting of the pillars was almost never observed during the course of the experiments and was therefore neglected.

### Preparation and transfer of the polystyrene film

**Figure 9:**
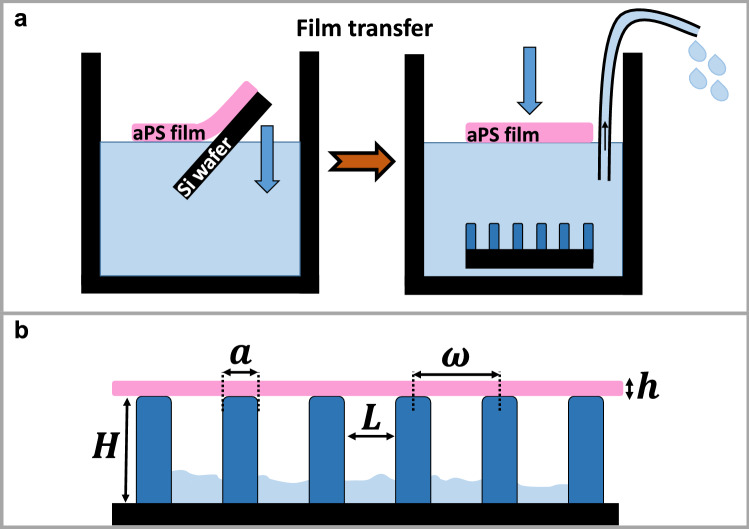
Schematic representation of the sample preparation. (**a**) Detaching the spin-coated polystyrene film from the substrate and transferring it on an array of pillars. (**b**) The film right after transferring (side view). Water may still be trapped in between the pillars.

Atactic polystyrene (aPS, *M*_*w*_ = 524 kg mol^−1^, *Đ* = 1.04, $T_{{\text{g}}}$ ≈ 100 °C) was dissolved in toluene. Thin films of aPS were obtained by spin coating the polymer solution directly onto silicon substrates that had previously undergone ultraviolet-ozone (UV-O_3_) treatment in order to make their surface hydrophilic by generating hydroxyl groups. When the sample was immersed in a bath of deionized water, water penetrates in between the film and the hydrophilic substrate. The polystyrene film then floats on the water surface. Subsequently, water was slowly removed with a syringe from the bath until the film touched the pillar array substrate, which had been placed at the bottom of the bath before (Fig. 9a).

After the transfer, the film was lying flat on top of the array of the deformable pillars. At this point, water may have been trapped between the film and the substrate (Fig. 9b). Therefore, in order to ensure that water was removed completely, experiments were performed after one day of keeping the sample in dry conditions at room temperature.

In the present study, we used films of two different thicknesses. For the examination of the early stage of dewetting and the measurement of the force field around a dewetting hole, we used 50 nm thick films, obtained by spin coating a 1% toluene solution of aPS at 2000 rpm. These films were transferred on a pillar array with a periodicity of $\omega$ = 10 μm. For the examination of the patterns that emerged at the later stages of dewetting, we used 400 nm thick films, obtained by spin coating a 2.5% toluene solution at 1000 rpm. These films were transferred on a pillar array with a periodicity of $\omega$ = 12 μm, i.e., 7 μm spacing between pillars, as these were the only substrates available at that time.

## Supplementary Information

Supplementary Information 1.

Supplementary Video 1.

Supplementary Video 2.

## Data Availability

The data that support the findings of this study are available from the corresponding author upon reasonable request.
