# Supplementary material for: Measurements of periodically perturbed dewetting force fields and their consequences on the symmetry of the resulting patterns
Source: Sci Rep. 2021 Jun 23;11:13149. doi: 10.1038/s41598-021-92544-x (PMC8222397; doi:10.1038/s41598-021-92544-x)
Supplement: Supplementary file 1 — Supplementary Information 1. [file 41598_2021_92544_MOESM1_ESM.pdf]

Supplementary information

for:

Measurements of periodically perturbed dewetting force fields and their consequences on the symmetry of the resulting patterns

Konstantinos Roumpos[1,2,3], Sarah Fontaine[4], Thomas Pfohl[1], Oswald Prucker[4], Jürgen Rühe[4] and Günter Reiter[1,2,3]

[1]Institute of Physics, University of Freiburg, Hermann-Herder-Straße 3a, 79104 Freiburg, Germany

[2]Freiburg Materials Research Center, Stefan-Meier-Straße 21, 79104 Freiburg, Germany

[3]Freiburg Center for Interactive Materials and Bioinspired Technologies (FIT), University of Freiburg, Georges-Köhler-Allee 105, 79110 Freiburg, Germany

[4]Department of Microsystems Engineering, University of Freiburg, Georges-Köhler-Allee 103, 79110 Freiburg, Germany

1. Movies of dewetting holes

Movies of dewetting holes as they grow larger and cause the pillars to deflect are given:

Supplementary Movie 1

4NP: Initially, hole was centered between 4 pillars

https://youtu.be/1Zv-EgKyWDo

Suplementary Movie 2

2NP: Hole starting to grow between 2 pillars

https://youtu.be/toja4kOZgKI

2. Groups of pillars for the 4NP and 2NP scenarios

	4NP		2NP	

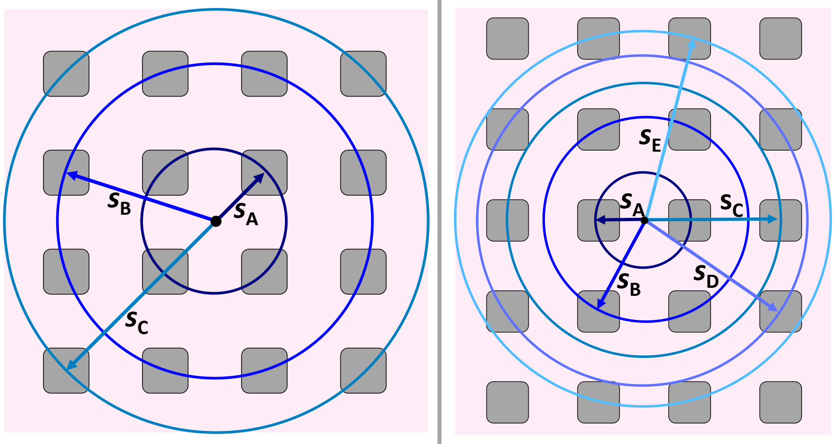

s_J	Distance	Pillars	Distance	Pillars
s_A	$\frac{1}{\sqrt{2}}\omega$	4	$\frac{1}{2}\omega$	2
s_B	$\sqrt{\frac{5}{2}}\omega$	8	$\frac{\sqrt{5}}{2}\omega$	4
s_C	$\frac{3}{\sqrt{2}}\omega$	4	$\frac{3}{2}\omega$	2
s_D	-	-	$\frac{\sqrt{13}}{2}\omega$	4
s_E	-	-	$\frac{\sqrt{17}}{2}\omega$	4

Supplementary Figure 1. Sketches of the top view of the samples for dewetting scenarios 4NP and 2NP. Each circle of radius s_J indicates the group J of pillars. The corresponding table includes the minimal distances s_J from the center of the dewetting hole as a function of the periodicity of the pattern, ω, and the number of pillars of each group.

3. Force acting on pillars as a function of the perimeter of a dewetting hole

The plot of the sum of the absolute value of the force on all pillars as a function of the perimeter P of the dewetting hole is given in Supplementary Fig. 2. In Fig. 4a of the main text, we see that the ratio $\Sigma|\vec{F}|/P$ reached a constant value after approximately $t_{\text{dew}} = 1500$ s of dewetting. According to Fig. 3a of the main text, $t_{\text{dew}} = 1500$ s corresponds to a dewetting perimeter $P \approx 75\ \mu\text{m}$. The slopes shown in Supplementary Fig. 2 were therefore calculated for $P > 75\ \mu\text{m}$.

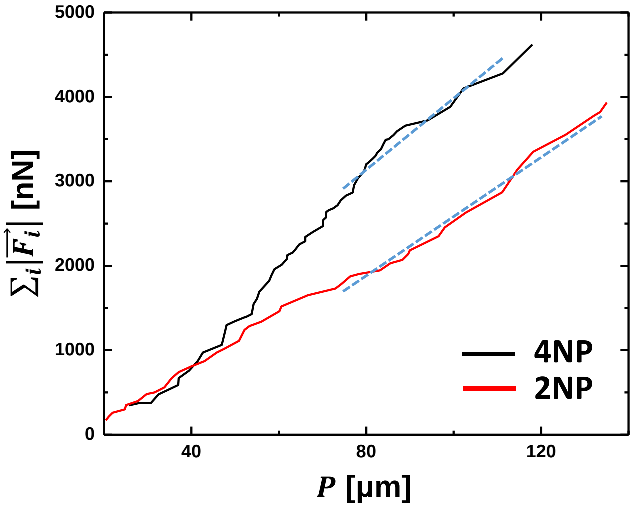

Supplementary Figure 2. The total force acting on all pillars as a function of the perimeter P of the dewetting hole. The slopes indicated with dashed lines in the graph were determined for $P > 75\ \mu\text{m}$. For 4NP holes, we obtained a value of 42 mN/m. For 2NP holes, we obtained a value of 36 mN/m.

4. The shape of the dewetting holes

The distorted shape of the hole can be better understood when plotting the diameters of the hole measured in the $x-$ and $y-$direction (d_x and d_y, respectively) and in the diagonal, 45° in respect to the $x-$ and $y-$directions (d_d) for 4NP holes, and the diameters in the $x-$ and $y-$direction for 2NP holes as a function of the dewetting time t_{dew} (Supplementary Fig. 3). For 4NP holes, we have symmetry in the $x-$ and $y-$directions (horizontally and vertically). Dewetting in these directions is not affected by the presence of pillars. The diameters measured in the $x-$ and $y-$directions were almost equal. Polymer flow was restricted in the direction of the 45° diagonal, where the contact line of the dewetting hole was pinned, i.e., directly in contact with the neighboring pillars. For 2NP holes, they grew freely in the $y-$direction. However, in the $x-$ direction, growth was restricted by the neighboring pillars.

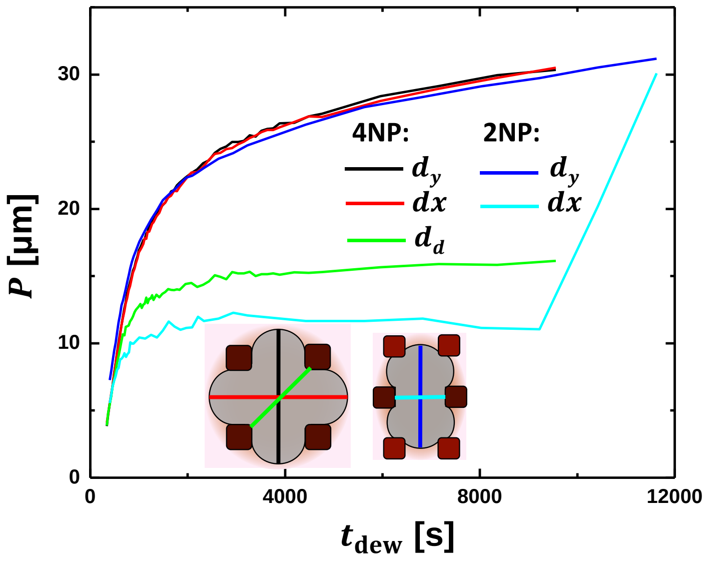

Supplementary Figure 3. Evolution of the shape of the dewetting holes. Diameters of the dewetting hole measured in the diagonal, $x-$ and $y-$directions. Notice the increase in $d_x(\text{2NP})$ for $t_{\text{dew}} > 9000\ s$, i.e., the unpinning of the nearest pillars to the dewetting hole.

5. Bar graphs showing the distribution of the total force (absolute value) for the various groups of pillars.

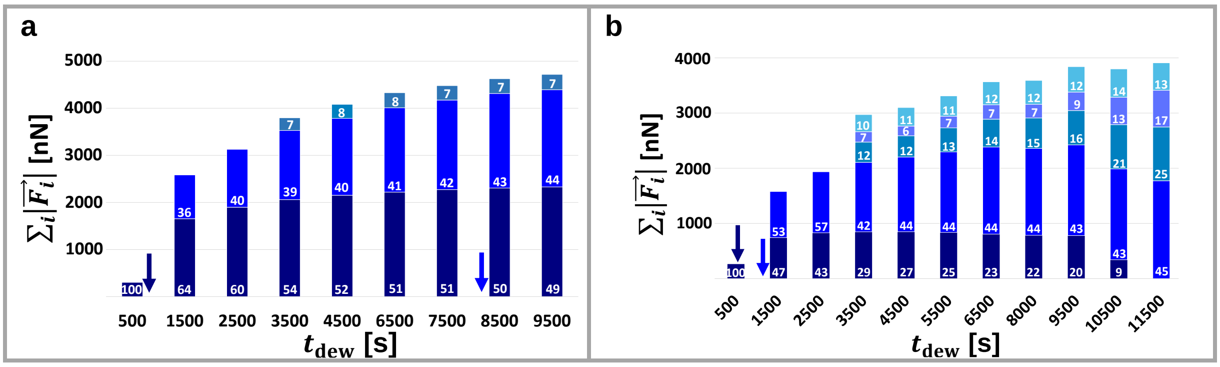

Supplementary Figure 4. Bar chart of the distribution of the total force to the groups of pillars. (**a**) for 4NP holes and (**b**) for 2NP holes. The different colors of the bars represent the various groups of pillars as in Fig. 2 of the main text. The arrows on the horizontal axis indicate the positions where the contact line of the dewetting holes first touched the pillars of group A and group B. The numbers inside the bars represent the percentage of the total force for each group of pillars.

6. Unpinning of pillars: a preliminary quantitative analysis

After unpinning of a pillar, the force that was acting upon this pillar just before unpinning was re-distributed to the neighboring pillars (for the example shown in Supplementary Fig. 5, the force is redistributed in total on 6 pillars of group C and group D). After unpinning, the contact line advances rapidly and eventually touches other neighboring pillars. We can support this claim by measuring the difference in the force on the neighboring pillars before and after unpinning and comparing it to the maximum force on the unpinned pillar.

In Supplementary Fig. 5, we have defined the “extra force” ΔF_A as the increase in the force acting on a pillar when the first pillar was unpinned (as can be seen in Supplementary Fig. 5, this unpinning event happened at a time between 9000 and 10500 s). We have labeled those pillars that showed the largest increase in force acting upon them with numbers from 0 to 5. For pillar 0, no “extra force” ΔF_A was detected after unpinning of the first pillar.

Therefore, it was not considered when we examined the unpinning of the first pillar. We obtained $\sum_{i=1}^{5} \Delta F_{A,i} = 0.43$ µN, which is close to the maximum pinning force $F = 0.46$ µN that was acting on the first pillar just before it became unpinned.

The “extra force” ΔF_B is the increase in the force acting on neighboring pillars when the second pillar became unpinned (as can be seen in Supplementary Fig. 5, this unpinning event happened at a time between 10500 and 11800 s). We obtained $\sum_{i=0}^{5} \Delta F_{B,i} = 0.41$ µN, which is close to the maximum pinning force $F = 0.42$ µN that was acting on the second pillar just before it became unpinned.

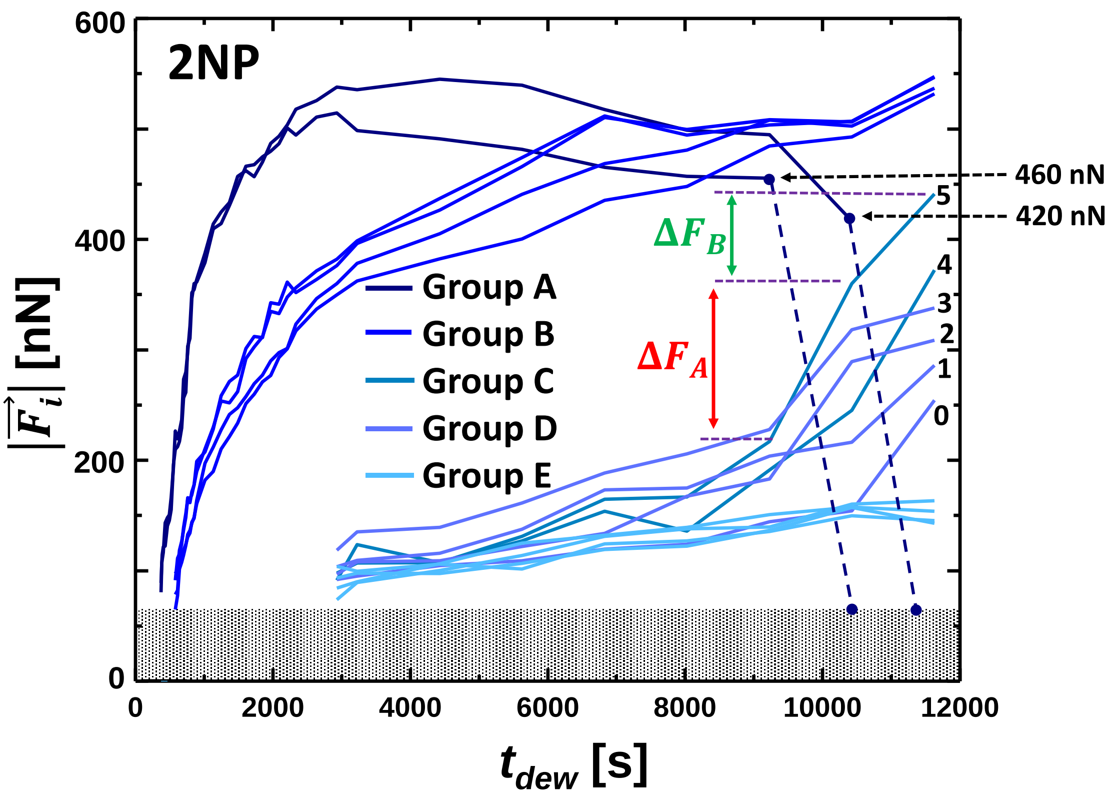

Supplementary Figure 5. Supplement to Figure 5b (Mapping the force fields) of the main text, including values of the “extra force” acting on neighboring pillars as a consequence of unpinning of a pillar.

6. Nucleation density of dewetting holes for 50 nm films

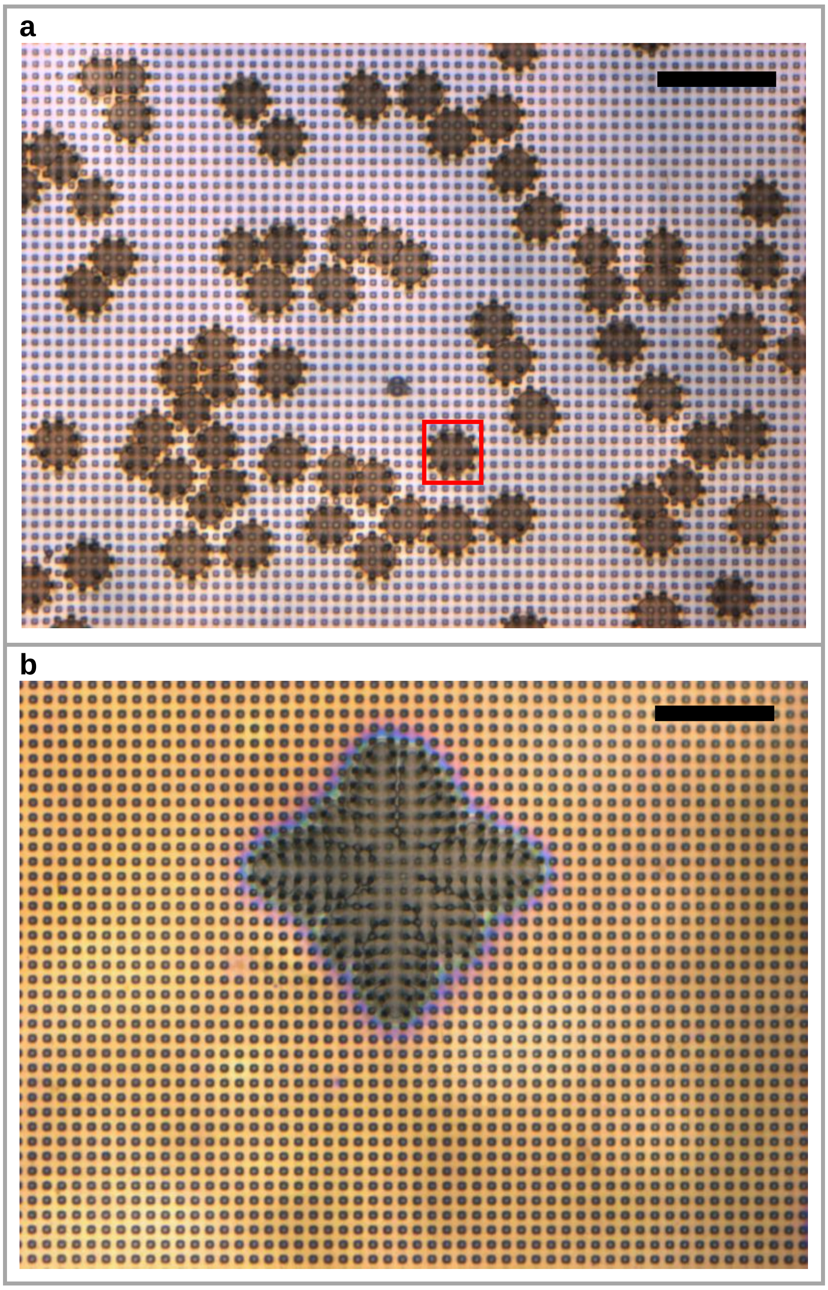

Supplementary Figure 6. Film thickness and nucleation density of dewetting holes. (**a**) Optical micrograph of a 50 nm film of polystyrene, taken after $t_{\text{dew}} \approx 12\ 000\ s$ of dewetting at 115 °C. The high nucleation density of dewetting holes caused frequent coalescence of dewetting holes, making further measurements unreliable. The red square indicates the area that was observed for the dewetting experiments of a 2NP hole. (**b**) Optical micrograph, using the same magnification as in (a), of a 400 nm film of polystyrene, taken after $t_{\text{dew}} \approx 1\ 500\ s$ of dewetting at 180 °C. Notice that there is only one hole in the same area as in (a). The length of the black bars represents 100 μm.

7. Analysis of optical micrographs

The pictures obtained from the microscope were analyzed using the ImageJ software. The dewetting holes were selected using the automatic outlining (wand) tool. This tool traces the edge of an object producing an outline of its selection. The wand tool allows to select a pixel as the chosen initial brightness value, which ranges from 0 to 255. The user also defines a tolerance value, also ranging from 0 to 255. The software then selects a contiguous area under the condition that all pixel values within that area must be within the range [*initial value* − *tolerance*] to [*initial value* + *tolerance*].

Dewetting holes were identified by setting an appropriate initial brightness value and a suitable tolerance value, which allowed the selection reach the boundary of the dewetting holes, i.e., the contact line. As a disadvantage of this method, pillars were included in the automated selection when they had similar color and brightness as the dewetting hole. This was often the case, when the contact line of the dewetting hole was touching and deforming pillars. In such cases, we have deselected these pillars using the rectangular selection tool of ImageJ. Selections for different tolerance values are shown in Supplementary Fig. 6. The presented dewetting hole is one for case A after $t_{\mathrm{dew}} = 1300\ \mathrm{s}$ of dewetting at $T_{\mathrm{dew}} = 115\ °\mathrm{C}$. Once a hole was properly identified, we calculated its area and perimeter by ImageJ.

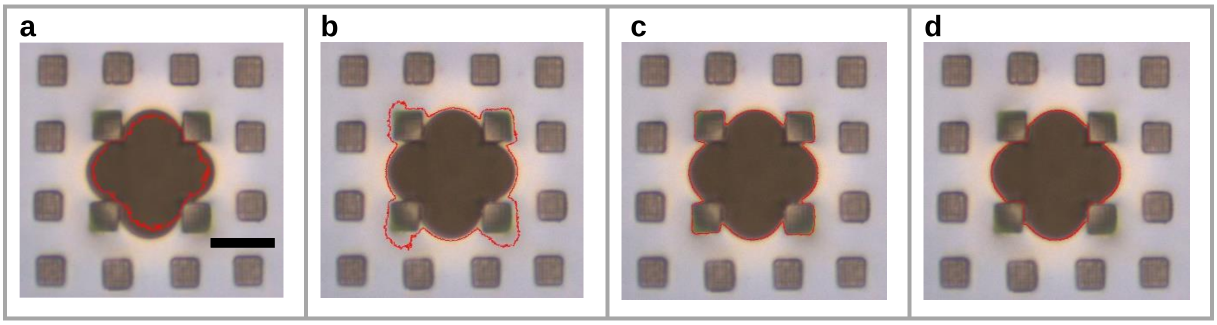

Supplementary Figure 7. Identifying a dewetting hole based on its brightness with ImageJ using the same initial value but different tolerance values. The selected area is enclosed by the red line. (**a**) Tolerance = 5; the selected area is smaller than the dewetting hole. Selection length: 113 μm; area: 271 μm^2. (**b**) Tolerance = 100; the selected area is larger than the dewetting hole. Selection length: 133 μm; area: 433 μm^2. (**c**) Tolerance = 50; the selected area

is bounded by the contact line of the dewetting hole but includes also the neighboring pillars. Selection length: 91 μm; area: 380 μm^2. (**d**) These pillars were deselected and the area inside the hole could now be measured correctly. Selection length: 71 μm; area: 298 μm^2. The length of the black bar in (**a**) represents 10 μm.
